# GNSS/SINS/DVL integrated navigation algorithm based on adaptive differential Kalman filtering

**DOI:** 10.1371/journal.pone.0342016

**Published:** 2026-02-05

**Authors:** Zhao Zhan, Changjian Liu, Kaidi Jin, Minzhi Xiang, Min Wang

**Affiliations:** 1 Information Engineering University, Zhengzhou, China; 2 Academy of Military Sciences, Beijing, China; University of New England School of Science and Technology, AUSTRALIA

## Abstract

The global navigation satellite system/strapdown inertial navigation system/doppler velocity logger (GNSS/SINS/DVL) integrated navigation system leverages the complementary advantages of its three subsystems to provide essential navigation information—such as attitude, velocity, and position—for carriers operating in marine environments. However, unmanned underwater vehicle (UUV) faces challenges like observation anomalies and dynamic model inaccuracies during dynamic maritime navigation and positioning. These issues make it difficult for the standard Kalman filter (KF) to cope with the complexities of the ocean environment, thereby reducing the accuracy of navigation parameter estimates. To address this, this study introduces an adaptive differential Kalman filtering (ADKF) method for processing integrated navigation data. Experimental results indicate that, compared with the KF, the proposed algorithm significantly enhances the accuracy and stability of parameter estimation, making it well-suited for post-processing integrated navigation data in complex marine settings.

## Introduction

As national maritime strategies evolve from focusing on coastal waters to embracing distant and global arenas, conflicts of interest stemming from ocean exploitation and utilization are intensifying, leading to a heightened demand for maritime target security. The rapid and precise acquisition of maritime target information has therefore become a strategic priority for major maritime powers vying for supremacy [1]. As a next-generation tool for ocean exploration, the unmanned underwater vehicle (UUV) now plays an increasingly pivotal role in underwater detection, maritime search, seabed mapping, and coordinated anti-submarine missions [2]. Accurate navigation parameters—including attitude, speed, and position—are critical to ensuring that UUV accomplishes its missions efficiently and navigate safely [3].

Due to the rapid attenuation of electromagnetic waves, radio frequency signals, and radio waves in seawater, signals from global navigation satellite system (GNSS)—commonly used for terrestrial and aerial navigation—become ineffective underwater [4]. Consequently, achieving precise underwater positioning of UUV has emerged as a major research focus and challenge [5]. The strapdown inertial navigation system (SINS) is typically employed as the primary navigation system for UUV, offering high autonomy, strong concealment, high reliability, robust dynamic performance, and a wide range of navigational parameters. However, SINS is prone to rapid error accumulation over time, necessitating the use of auxiliary sensors for error correction [6,7]. Common auxiliary sensors include the doppler velocity log (DVL), pressure sensors (PS), underwater acoustic positioning systems, and geophysical field navigation systems [8]. Nonetheless, acoustic positioning technologies such as long baseline (LBL) and ultra-short baseline (USBL) require the pre-deployment of expensive underwater acoustic arrays and lack concealment [9]. Geophysical field navigation systems, such as gravity and geomagnetic matching, require prior data collection and the establishment of specialized databases, limiting their use in uncharted waters [10]. Pressure sensors provide high-precision depth information by measuring underwater pressure, effectively compensating for SINS’s accumulated errors. The DVL transmits ultrasonic waves towards the seafloor through acoustic transducers and receives their reflections. By utilizing the doppler frequency shift between the transmitted and reflected waves, it calculates the velocity of the vehicle relative to the water mass or the seafloor. In particular, a four-beam DVL configures four transducers in a Janus configuration, which additionally eliminates velocity measurement errors induced by the vehicle’s heave, roll, and pitch motions. As a result, the DVL/SINS integrated navigation system has become the predominant method for autonomous underwater navigation of UUV [11]. However, UUV is required to operate not only underwater but also on the surface. When operating on the surface, UUV typically utilizes a GNSS/SINS/DVL integrated navigation system as its primary method for autonomous navigation. This system generally employs KF for data processing, and based on the configuration of the sensors, the level of information exchange, and the degree of integration, the GNSS/SINS/DVL system can be classified into loosely coupled and tightly coupled types [12]. Given the limitations in computational resources and the fact that most DVLs only provide three-dimensional velocity outputs, loosely coupled systems have received extensive research attention. During surface navigation, UUV equipped with GNSS/SINS/DVL system may experience significant errors in GNSS observations due to abnormal disturbances [13,14]. Additionally, the complex marine environment and sensor characteristics may cause DVL measurements to exhibit time-varying noise, non-Gaussian noise, and gross errors [15]. These factors can sometimes result in suboptimal filter performance, thus reducing the accuracy of the GNSS/SINS/DVL integrated navigation system. Since the performance of a filter is affected by the accuracies of the stochastic model and the functional model, only when both of them are reliable can the KF obtain the optimal estimation of state parameters. However, in practical applications, there exist errors in the state equation and measurement equation. The measurement noise and process noise described by the stochastic model are usually derived from empirical values or empirical models, which fail to accurately reflect the actual noise level of the current system. This not only degrades the accuracy of KF but may even lead to filter divergence in severe cases.

To enhance the performance robustness of filtering algorithms in complex practical scenarios, the academic community has conducted extensive and in-depth research on adaptive filtering and robust filtering [16–18]. In the field of real-time estimation of system noise covariance matrices, relevant studies have proposed various effective algorithms, among which the Sage-Husa filter has been widely applied due to its concise principle and strong practicality [19,20]. This method realizes the recursive estimation of the process noise covariance matrix at the current epoch through the filtering innovation or residual sequence within a sliding fixed window. Although the real-time estimation of the noise covariance matrix can maintain the consistency between the predicted residuals and theoretical statistical characteristics, the filtering performance of such methods is highly dependent on the accuracy of the functional model. Its core limitation lies in the strong coupling relationship between system noise estimation and state parameter prediction: when there are modeling errors in the functional model, these errors will propagate to the noise covariance matrix estimation process through the coupling mechanism, thereby reducing the filtering estimation accuracy. To address this problem, scholars have proposed noise variance estimation algorithms based on redundant measurements for navigation systems with redundant measurement characteristics [21]. Such methods effectively improve the stability of filtering results by decoupling the association between noise covariance matrix estimation and state estimation errors. Meanwhile, for scenarios where the state model has significant errors, researchers have proposed fading filtering and adaptive filtering methods. These two types of algorithms dynamically adjust the predicted state covariance matrix by introducing a fading factor or an adaptive factor, respectively, thereby suppressing the adverse effects of state model errors on filtering results [22,23]. The core of adaptive filtering lies in the construction of the adaptive factor; therefore, the academic community has carried out extensive research on the solution methods of the adaptive factor and proposed various discriminant statistics for adaptive factor design, such as state inconsistency statistics, variance component ratio statistics, and predicted residual statistics [24]. To achieve the coordinated adaptive processing of model errors and observation errors, and effectively solve the coupled influence problem of observation outliers and abnormal motion disturbances in complex scenarios, this paper organically integrates adaptive filtering and robust filtering, and proposes an adaptive differential Kalman filtering (ADKF) algorithm. This algorithm can accurately identify and separate the coupled influences of observation outliers and abnormal motion disturbances on positioning results, providing technical support for high-precision state estimation in complex scenarios. To verify the effectiveness and superiority of the proposed algorithm, actual dynamic test data are used for simulation verification. The test results show that the proposed ADKF algorithm can adaptively adjust the filtering parameters based on real-time measurement information, realize the effective suppression of model errors and observation errors, significantly enhance the resistance of the filtering algorithm to the influences of observation outliers and abnormal motion disturbances on positioning results, and thus improve the stability and reliability of state estimation in the integrated navigation system.

### Integrated navigation system

The sensors within the GNSS/SINS/DVL integrated navigation system include a GNSS receiver, an inertial measurement unit (IMU), and a DVL [12]. The GNSS receiver determines the receiver’s position, velocity, and heading by processing satellite signals to output pseudorange, pseudorange rate, and carrier phase measurements. The IMU, comprising a three-axis gyroscope and a three-axis accelerometer, provides direct measurements of angular velocity and specific forces in the Earth-centered inertial frame. The DVL emits ultrasonic waves underwater using a transducer and receives the echoes, thereby directly measuring the UUV’s three-dimensional velocity relative to the seabed or the water layer [11]. Given that these three sensors operate at different sampling frequencies, synchronizing the timestamps of their output data to a unified time reference is crucial to prevent system divergence during data fusion due to timing discrepancies. Beyond temporal synchronization, spatial alignment is also necessary because sensor installation deviations on the UUV introduce lever arm errors [5]. During system initialization, spatial synchronization is used to determine the rotations and translations between the coordinate systems of each sensor. The data fusion algorithms for GNSS/SINS/DVL integrated navigation system are mainly divided into three categories: centralized fusion algorithms, parallel fusion algorithms, and sequential fusion algorithms. In this paper, a sequential fusion algorithm tailored for linear systems is proposed, which employs an adaptive factor method to overcome the issues of asymmetry and prior sensitivity inherent in traditional sequential fusion. Based on the output frequencies, the algorithm sequentially inputs measurement data from each sensor into the fusion subsystem, processes them one by one, and ultimately outputs the integrated navigation solution.

### Filtering model

#### Kalman filter.

In the GNSS/SINS/DVL integrated navigation system, the SINS acts as the primary navigation system. The differences in position between GNSS and SINS, as well as velocity differences between DVL and SINS, are used as measurement vectors for data fusion via the KF. To simplify system design, navigation parameter errors are selected as the state vector of the filter, and the estimated errors are applied to correct the SINS output. Since the accuracy of DVL velocity measurements is affected by water temperature, salinity, depth, and acoustic frequency, the DVL observations are typically modeled as follows in practical applications [15]:


$${\tilde{v}}^{d}=(1+\delta k)v^{d}+w^{d}$$ (1)

In the Eq. (1), δk denotes the scale factor modeled as a constant value, $v^{d}$ represents the three-dimensional velocity, and $w^{d}$ is Gaussian white noise.

The state parameters of the integrated navigation system include: (1) SINS navigation parameter errors, such as misalignment angles ϕ, velocity errors $\delta v^{n}$, position errors δp, constant gyroscope bias $\varepsilon^{b}$, and constant accelerometer bias $\nabla^{b}$; (2) DVL error parameters, including installation angle error η, lever arm error δl, scale factor error δk, and time synchronization error δt. These together form a 23-dimensional state vector:


$$x={\left[\begin{array}{cccccccc} @cccccccc\phi^{\text{T}} & {\left(\delta v^{n}\right)}^{\text{T}} & {\left(\delta p\right)}^{\text{T}} & {\left(\varepsilon^{b}\right)}^{\text{T}} & {\left(\nabla^{b}\right)}^{\text{T}} & \eta^{\text{T}} & \delta l^{\text{T}} & \delta k \end{array}\delta t\right]}^{\text{T}}$$ (2)

In practical applications, SINS navigation errors accumulate over time. The error propagation equation reveals the pattern of error propagation and serves as a fundamental basis for research on inertial-based integrated navigation systems. The SINS error update equation is as follows.

(1) Attitude error equation


$$\left\{ \begin{array}{c} {\dot{\phi }}_{E}=(\omega_{U}+\frac{v_{E}tanL}{R_{Nh}})\phi_{N}-(\omega_{N}+\frac{v_{E}}{R_{Nh}})\phi_{U}-\frac{\text{1}}{R_{Mh}}\delta v_{N}+\frac{v_{N}}{R_{Mh}^{\text{2}}}\delta h-\varepsilon_{E}\\ {\dot{\phi }}_{N}=-(\omega_{U}+\frac{v_{E}tanL}{R_{Nh}})\phi_{E}-\frac{v_{N}}{R_{Mh}}\phi_{U}+\frac{\text{1}}{R_{Nh}}\delta v_{E}-\omega_{U}\delta L-\frac{v_{E}}{R_{Mh}^{\text{2}}}\delta h-\varepsilon_{N}\\ {\dot{\phi }}_{U}=(\omega_{N}+\frac{v_{E}}{R_{Nh}})\phi_{E}+\frac{v_{N}}{R_{Mh}}\phi_{N}+\frac{tanL}{R_{Nh}}\delta v_{E}+(\omega_{N}+\frac{v_{E}{sec}^{\text{2}}L}{R_{Nh}})\delta L-\frac{v_{E}tanL}{R_{Nh}^{\text{2}}}\delta h-\varepsilon_{U} \end{array}\right.$$ (3)

Rewritten in matrix form, it can be obtained


$$\dot{\phi }=\phi \times \omega_{in}^{n}+\delta \omega_{in}^{n}-C_{b}^{n}\delta \omega_{ib}^{b}$$ (4)

According to the property of vector cross product, it is rewritten as


$$\dot{\phi }=M_{aa}\phi +M_{av}\delta v^{n}+M_{ap}\delta p^{n}-C_{b}^{n}\delta \omega_{ib}^{b}$$ (5)

(2) Velocity error equation


$$\left\{ \begin{array}{c} \delta {\dot{v}}_{E}=-f_{U}\phi_{N}+f_{N}\phi_{U}+\frac{v_{N}tanL-v_{U}}{R_{Nh}}\delta v_{E}+(2\omega_{U}+\frac{v_{E}tanL}{R_{Nh}})\delta v_{N}-(2\omega_{N}+\frac{v_{E}}{R_{Nh}})\delta v_{U}\\ \text{\textbackslash{}hspace\{3em}\}+\left[2(v_{N}\omega_{N}+v_{U}\omega_{U})+\frac{v_{E}v_{N}{sec}^{\text{2}}L}{R_{Nh}}\right]\delta L+\frac{v_{E}(v_{U}-v_{N}tanL)}{R_{Nh}^{\text{2}}}\delta h+\nabla_{E}\\ \delta {\dot{v}}_{N}=f_{U}\phi_{E}-f_{E}\phi_{U}-2(\omega_{U}+\frac{v_{E}tanL}{R_{Nh}})\delta v_{E}-\frac{v_{U}}{R_{Mh}}\delta v_{N}-\frac{v_{N}}{R_{Mh}}\delta v_{U}\\ \text{\textbackslash{}hspace\{1em}\}-v_{E}(2\omega_{N}+\frac{v_{E}{sec}^{\text{2}}L}{R_{Nh}})\delta L+(\frac{v_{N}v_{U}}{R_{Mh}^{\text{2}}}+\frac{v_{E}^{\text{2}}tanL}{R_{Nh}^{\text{2}}})\delta h+\nabla_{N}\\ \delta {\dot{v}}_{U}=-f_{N}\phi_{E}+f_{E}\phi_{N}+2(\omega_{N}+\frac{v_{E}}{R_{Nh}})\delta v_{E}+\frac{2v_{N}}{R_{Mh}}\delta v_{N}\\ \text{\textbackslash{}hspace\{1em}\}-\left[2\omega_{U}v_{E}+g_{e}sin2L(\beta -4\beta_{\text{1}}cos2L)\right]\delta L-(\frac{v_{E}^{\text{2}}}{R_{Nh}^{\text{2}}}+\frac{v_{N}^{\text{2}}}{R_{Mh}^{\text{2}}}-\beta_{\text{2}})\delta h+\nabla_{U} \end{array}\right.$$ (6)

Rewritten in matrix form, it can be obtained


$$\delta {\dot{v}}^{n}=\left(C_{b}^{n}f^{b}\right)\times \phi +C_{b}^{n}\delta f^{b}-\left(2\omega_{ie}^{n}+\omega_{en}^{n}\right)\times \delta v^{n}+v^{n}\times \left(2\delta \omega_{ie}^{n}+\delta \omega_{en}^{n}\right)$$ (7)

In the Eq. (7), $v^{n}={\left[\begin{array}{ccc} v_{E}^{n} & v_{N}^{n} & v_{U}^{n} \end{array}\right]}^{\text{T}}$ denotes the velocity vector of the SINS, where $v_{E}^{n}$, $v_{N}^{n}$ and $v_{U}^{n}$ represent the eastward, northward, and upward velocities of the SINS, respectively. $C_{b}^{n}$ denotes the attitude matrix of the body frame relative to the navigation frame, $f^{b}$ represents the specific force measurement of the accelerometer, ϕ is the misalignment angle error, $\delta f^{b}$ denotes the accelerometer measurement error, $\omega_{ie}^{n}$ is the Earth’s rotational angular velocity, $\omega_{en}^{n}$ is the angular velocity of the navigation frame, $\delta v^{n}$ represents the velocity error.

According to the property of vector cross product, it is rewritten as


$$\delta {\dot{v}}^{n}=M_{va}\phi +M_{vv}\delta v^{n}+M_{vp}\delta p^{n}+C_{b}^{n}\delta f_{\text{sf}}^{b}$$ (8)

(3) Position error equation


$$\left\{ \begin{array}{c} \delta \dot{L}=\frac{\text{1}}{R_{Mh}}\delta v_{N}-\frac{v_{N}}{R_{Mh}^{\text{2}}}\delta h\\ \delta \dot{\lambda }=\frac{secL}{R_{Nh}}\delta v_{E}+\frac{v_{E}secLtanL}{R_{Nh}}\delta L-\frac{v_{E}secL}{R_{Nh}^{\text{2}}}\delta h\\ \delta \dot{h}=\delta v_{U} \end{array}\right.$$ (9)

Rewritten in matrix form, it can be obtained


$$\delta {\dot{p}}^{n}=M_{pv}\delta v^{n}+M_{pp}\delta p^{n}$$ (10)

The specific forms of $M_{pv}$ and $M_{pp}$ are


$$\begin{array}{cc} M_{pv}=\left[\begin{array}{ccc} 0 & 1/R_{Mh} & 0\\ secL/R_{Nh} & 0 & 0\\ 0 & 0 & 1 \end{array}\right], & M_{pp}=\left[\begin{array}{ccc} 0 & 0 & -v_{N}^{n}/R_{Mh}^{\text{2}}\\ v_{E}^{n}secLtanL/R_{Nh} & 0 & -v_{E}^{n}secL/R_{Nh}^{\text{2}}\\ 0 & 0 & 0 \end{array}\right] \end{array}$$ (11)

The state space model of GNSS/SINS/DVL integrated navigation with velocity and position error as the direction-finding quantities is constructed as follows:


$$\dot{X}=FX+GW^{b}$$ (12)

In the Eq. (12), F is the one-step state transition matrix, G is the system noise distribution matrix, H is the measurement matrix, $W^{b}$ represents the system noise vector, and V denotes the measurement noise vector. Both $W^{b}$ and V are zero-mean Gaussian white noise vectors and are mutually uncorrelated. The specific forms of these matrices are as follows:


$$F=\left[\begin{array}{c} \begin{array}{cccccc} M_{aa} & M_{av} & M_{ap} & -C_{b}^{n^{\prime }} & {\text{0}}_{3\times 3} & {\text{0}}_{3\times 8}\\ M_{va} & M_{vv} & M_{vp} & {\text{0}}_{3\times 3} & C_{b}^{n^{\prime }} & {\text{0}}_{3\times 8}\\ {\text{0}}_{3\times 3} & M_{pv} & M_{pp} & {\text{0}}_{3\times 3} & {\text{0}}_{3\times 3} & {\text{0}}_{3\times 8} \end{array}\\ \begin{array}{cccccc} & & & & & {\text{0}}_{14\times 23} \end{array} \end{array}\right]$$ (13)

$$\begin{array}{cc} G=\left[\begin{array}{c} \begin{array}{cc} -C_{b}^{n^{\prime }} & {\text{0}}_{3\times 3}\\ {\text{0}}_{3\times 3} & C_{b}^{n} \end{array}\\ {\text{0}}_{17\times 6} \end{array}\right], & W^{b}=\left[\begin{array}{c} w_{g}^{b}\\ w_{a}^{b} \end{array}\right] \end{array}$$ (14)

In the loosely coupled GNSS/SINS integration scheme, the measurement vector is typically constructed from the differences in position and velocity provided by GNSS and SINS. The measurement equation can thus be expressed as follows:


$$Z_{\text{1}}=\left[\begin{array}{c} {\tilde{v}}_{INS}^{n}-{\tilde{v}}_{GNSS}^{n}\\ {\tilde{p}}_{INS}-{\tilde{p}}_{GNSS} \end{array}\right]=H_{\text{1}}X+V_{\text{1}}$$ (15)

In the Eq. (15), $Z_{\text{1}}$ represents the measurement vector of the loosely coupled system, $H_{\text{1}}$ is the measurement matrix, and $V_{\text{1}}$ denotes the measurement noise. The specific forms of $H_{\text{1}}$ and $V_{\text{1}}$ are as follows:


$$H_{\text{1}}=\left[\begin{array}{c} {\text{0}}_{3\times 3}\;\;\;I_{3\times 3}\;\;\;\text{\textbackslash{}textrm\{0}{\}}_{3\times 3}\;\;\;\text{\textbackslash{}textrm\{0}{\}}_{3\times 7}\;\;\;\text{-}C_{b}^{n}\text{(}w_{eb}^{b}\times \text{)}\;\;\;a^{n}\\ {\text{0}}_{3\times 3}\;\;\;{\text{0}}_{3\times 3}\;\;\;I_{3\times 3}\;\;\;\text{\textbackslash{}textrm\{0}{\}}_{3\times 7}\;\;\;\text{-}M_{pv}C_{b}^{n}\;\;\;M_{pv}v_{INS}^{n} \end{array}\right],V_{\text{1}}=\left[\begin{array}{c} V_{v}\\ V_{p} \end{array}\right]$$ (16)

In the loosely coupled DVL/SINS integration, the DVL provides the UUV’s three-dimensional body-frame velocity to correct SINS errors. Taking into account the DVL’s installation misalignment angle η and lever-arm error δl, the DVL velocity Eq. (1) can be rewritten as follows:


$${\tilde{v}}^{d}=\left(1+\delta k\right)C_{b}^{d}\left(v^{b}+\omega_{eb}^{b}\times \delta l\right)+w^{d}$$ (17)

In the Eq. (17), $\omega_{eb}^{b}=\omega_{ib}^{b}-C_{n}^{b}\omega_{ie}^{n}$ denotes the projection of the angular velocity of the body frame relative to the e frame onto the b frame. $C_{b}^{d}$ represents the direction cosine matrix corresponding to the DVL installation misalignment angle; $v^{b}$ represents the speed of the UUV carrier system. When the UUV undergoes attitude maneuvers, $\omega_{eb}^{b}\gg C_{n}^{b}\omega_{ie}^{n}$, it can be approximated that $\omega_{eb}^{b}\approx \omega_{ib}^{b}$.

The body-frame velocity can be expressed in terms of SINS navigation parameters and their errors.


$$\begin{array}{c} v^{b}=C_{n}^{b}v^{n}=C_{n^{\prime }}^{b}C_{n}^{n^{\prime }}\left({\tilde{v}}^{n}-\delta v^{n}\right)\approx C_{n^{\prime }}^{b}\left(I-\phi \times \right)\left({\tilde{v}}^{n}-\delta v^{n}\right)\\ \begin{array}{cc} & \approx \end{array}C_{n^{\prime }}^{b}{\tilde{v}}^{n}+C_{n^{\prime }}^{b}\left({\tilde{v}}^{n}\times \right)\phi -C_{n^{\prime }}^{b}\delta v^{n} \end{array}$$ (18)

Substituting Eq. (18) into Eq. (17), neglecting second-order small quantities, and simplifying, we obtain the following result.


$$\begin{array}{c} {\tilde{v}}^{d}=v^{b}-v^{b}\times \eta +\omega_{eb}^{b}\times \delta l+v^{b}\delta k+w^{d}\\ \begin{array}{cc} & \approx \end{array}C_{n^{\prime }}^{b}{\tilde{v}}^{n}+C_{n^{\prime }}^{b}\left({\tilde{v}}^{n}\times \right)\phi -C_{n^{\prime }}^{b}\delta v^{n}-\left(C_{n^{\prime }}^{b}{\tilde{v}}^{n}\right)\times \eta +\omega_{eb}^{b}\times \delta l+\left(C_{n^{\prime }}^{b}{\tilde{v}}^{n}\right)\delta k+w^{d} \end{array}$$ (19)

By rearranging the terms in Eq. (19), the measurement equation can be obtained.


$$Z_{\text{2}}=C_{n^{\prime }}^{b}{\tilde{v}}^{n}-{\tilde{v}}^{d}=H_{\text{2}}X+v$$ (20)

In the Eq. (20), $Z_{\text{2}}$ is the measurement vector of the loosely coupled system; $H_{\text{2}}$ is the measurement matrix of the loosely coupled system; and $v=w^{d}$ denotes the measurement noise. The specific form of $H_{\text{2}}$ is as follows:


$$H_{\text{2}}=\left[\begin{array}{cccccc} -C_{n^{\prime }}^{b}\left({\tilde{v}}^{n}\times \right) & C_{n^{\prime }}^{b} & {\text{0}}_{3\times 10} & \left(C_{n^{\prime }}^{b}{\tilde{v}}^{n}\right)\times & -\left(\omega_{eb}^{b}\times \right) & -C_{n^{\prime }}^{b}{\tilde{v}}^{n} \end{array}\right]$$ (21)

### Adaptive differential Kalman filter

During UUV navigation, the complex marine environment often causes the statistical characteristics of GNSS measurement noise to deviate from the ideal Gaussian distribution assumption [8]. System measurement noise is easily disturbed or contaminated, leading to degraded filtering performance. DVL measurements can also exhibit anomalies, including slow drifts, sudden changes in noise, and partial beam loss. These factors introduce shortcomings in the GNSS/SINS/DVL integrated navigation system, ultimately undermining the accuracy of UUV navigation and positioning. To address these challenges, this section applies ADKF to the state estimation of the UUV’s GNSS/SINS/DVL integrated navigation system, with the goal of reducing the impact of contaminated measurement noise and enhancing the overall robustness of the integrated navigation approach.

The system state-space model is given by Eq. (12) and Eq. (15). Assuming that the observation residual vector is $V_{k}$ and the state prediction vector is ${\overset{―}{X}}_{k}$,the observation error equation and the state prediction equation are denoted as


$$\left\{ \begin{array}{c} V_{k}=H_{k}{\hat{X}}_{k}-Z_{k}\\ {\overset{―}{X}}_{k}=F_{k,k-1}{\hat{X}}_{k-1} \end{array}\right.$$ (22)

In the Eq. (22), ${\hat{X}}_{k}$ and ${\hat{X}}_{k-1}$ represent the state estimation vectors at time instants $t_{k}$ and $t_{k-1}$, respectively.

To suppress the influence of abnormal measurement information and abnormal disturbances of the dynamic model on the navigation solution, the principle of ADKF is shown in Eq. (23).


$$\Omega \left(k\right)=V_{k}^{\text{T}}{\overset{―}{\Sigma }}_{k}^{-1}V_{k}+\alpha_{k}V_{{\overset{―}{X}}_{k}}^{T}\Sigma_{{\overset{―}{X}}_{k}}^{-1}V_{{\overset{―}{X}}_{k}}=min$$ (23)

In the Eq. (23), ${\overset{―}{\Sigma }}_{k}^{-1}$ denotes the equivalent weight matrix of the measurement vector, which serves as an adaptive estimate of the measurement covariance matrix. $\alpha_{k}$ ($0<\alpha_{k}\leq 1$) is an adaptive factor; when the dynamic model encounters anomalies, $\alpha_{k}$ decreases, thereby controlling the impact of abnormal errors on state parameter estimation. When $\alpha_{k}$ = 1, the adaptive filter is equivalent to the KF. Additionally, $\sum_{{\overset{―}{X}}_{k}}^{-1}$ functions as the equivalent weight matrix for the predicted state vector ${\overset{―}{X}}_{k}$.

By differentiating Eq. (23) and setting the result to zero, we obtain the following.


$$\frac{d\Omega \left(k\right)}{d{\hat{X}}_{k}}=2V_{k}^{\text{T}}{\overset{―}{\Sigma }}_{k}^{-1}H_{k}+2\alpha_{k}V_{{\overset{―}{X}}_{k}}^{\text{T}}\Sigma_{{\overset{―}{X}}_{k}}^{-1}=0$$ (24)

After further simplification, we obtain the following result.


$$H_{k}^{\text{T}}{\overset{―}{\Sigma }}_{k}^{-1}V_{k}+\alpha_{k}\Sigma_{{\overset{―}{X}}_{k}}^{-1}V_{{\overset{―}{X}}_{k}}=0$$ (25)

Substituting $V_{{\overset{―}{X}}_{k}}={\hat{X}}_{k}-{\overset{―}{X}}_{k},V_{k}=H_{k}{\hat{X}}_{k}-Z_{k}$ into Eq. (25), we obtain


$$\left(H_{k}^{\text{T}}{\overset{―}{\Sigma }}_{k}^{-1}H_{k}+\alpha_{k}\Sigma_{{\overset{―}{X}}_{k}}^{-1}){\hat{X}}_{k}=(H_{k}^{\text{T}}{\overset{―}{\Sigma }}_{k}^{-1}Z_{k}+\alpha_{k}\Sigma_{{\overset{―}{X}}_{k}}^{-1}{\overset{―}{X}}_{k}\right)$$ (26)

Eq. (26) can be equivalently expressed as


$${\hat{X}}_{k}={\overset{―}{X}}_{k}+{\overset{―}{K}}_{k}\left(Z_{k}-H_{k}{\overset{―}{X}}_{k}\right)$$ (27)

In the Eq. (27), ${\overset{―}{K}}_{k}$ represents the adaptive robust filtering gain.


$${\overset{―}{K}}_{k}=\frac{\text{1}}{\alpha_{k}}\Sigma_{{\overset{―}{X}}_{k}}H_{k}^{\text{T}}{\left(\frac{\text{1}}{\alpha_{k}}H_{k}\Sigma_{{\overset{―}{X}}_{k}}H_{k}^{\text{T}}+{\overset{―}{\Sigma }}_{k}\right)}^{-1}$$ (28)

According to the covariance propagation law, the a posteriori covariance matrix of the state estimate vector ${\hat{X}}_{k}$ is shown in Eq. (29).


$$\begin{array}{c} \Sigma_{{\hat{X}}_{k}}=\left(I-{\overset{―}{K}}_{k}H_{k}\right)\Sigma_{{\overset{―}{X}}_{k}}{\left(I-{\overset{―}{K}}_{k}H_{k}\right)}^{\text{T}}+{\overset{―}{K}}_{k}{\overset{―}{\Sigma }}_{k}{\overset{―}{K}}_{k}^{\text{T}}\\ =\frac{\text{1}}{\alpha_{k}}\left(I-{\overset{―}{K}}_{k}H_{k}\right)\Sigma_{{\overset{―}{X}}_{k}} \end{array}$$ (29)

In ADKF, the measurement noise covariance matrix is determined using robust estimation methods, and adaptive factors are employed to adjust the information weights of the measurement and state equations. This approach effectively controls the influence of measurement outliers and dynamic model noise anomalies on state estimation. The equivalent weight matrix for the measurement vector can be calculated using either the Huber function or IGG-type schemes [17]. In this study, three statistical metrics for learning dynamic model errors were developed: the state inconsistency statistic, the prediction residual statistic, and the variance component ratio statistic [24].

(1)The state inconsistency statistic is constructed using the a posteriori estimate of the state parameter ${\hat{X}}_{k}$ and the one-step predicted value ${\overset{―}{X}}_{k}$.


$$\Delta {\tilde{X}}_{k}=\|{\hat{X}}_{k}-{\overset{―}{X}}_{k}\|/\sqrt{\text{tr}\left(\Sigma_{{\overset{―}{X}}_{k}}\right)}$$ (30)

(2)The innovation vector ${\overset{―}{V}}_{k}$ reflects the error between the one-step predicted state ${\overset{―}{X}}_{k}$ and the measurement $Z_{k}$. This innovation vector is used to construct the prediction residual statistic.


$$\Delta V_{k}={\overset{―}{V}}_{k}^{\text{T}}{\overset{―}{V}}_{k}/\sqrt{\text{tr}\left(\Sigma_{{\overset{―}{V}}_{k}}\right)}$$ (31)

(3)Based on measurement information and predictions from the dynamic model, the variance components of the one-step predicted state ${\overset{―}{X}}_{k}$ and the measurement $Z_{k}$ can reflect both measurement and model accuracy. An error discrimination statistic is constructed using the ratio of these variance components. The Helmert variance component estimation formulas for ${\overset{―}{X}}_{k}$ and $Z_{k}$ are as follows:


$$\sigma_{0,k}^{\text{2}}=\frac{V_{k}^{\text{T}}P_{k}V_{k}}{r_{k}},\sigma_{0,{\overset{―}{X}}_{k}}^{\text{2}}=\frac{V_{{\overset{―}{X}}_{k}}^{\text{T}}P_{{\overset{―}{X}}_{k}}V_{{\overset{―}{X}}_{k}}}{r_{{\overset{―}{X}}_{k}}}$$ (32)

Construct the error discrimination statistic using the ratio of variance components:


$$S_{k}=\frac{\sigma_{0,{\overset{―}{X}}_{k}}^{\text{2}}}{\sigma_{0,k}^{\text{2}}}\approx \frac{V_{{\overset{―}{X}}_{k}}^{\text{T}}P_{{\overset{―}{X}}_{k}}V_{{\overset{―}{X}}_{k}}}{r_{{\overset{―}{X}}_{k}}\sigma_{0,{\overset{―}{X}}_{k}}^{\text{2}}}$$ (33)

The determination of the adaptive factor $\alpha_{k}$ can be accomplished using various function models, such as two-segment, three-segment, or exponential function models. In the discussion of the adaptive factor, the specific forms are illustrated using the state inconsistency statistic $\Delta {\tilde{X}}_{k}$ as an example.

(1) The two-segment function model introduces a hyperparameter as a threshold. When the error discrimination statistic is less than the threshold, a = 1*a* = 1, and KF is performed. If the error discrimination statistic exceeds the threshold, the contribution of the state prediction model to state estimation is reduced. The functional form is as follows:


$$\alpha_{k}=\left\{ \begin{array}{cc} 1 & \left|\Delta {\tilde{X}}_{k}\right|\leq c\\ \frac{c}{\left|\Delta {\tilde{X}}_{k}\right|} & \left|\Delta {\tilde{X}}_{k}\right|>c \end{array}\right.$$ (34)

In the Eq. (34), c is a constant with a value of 1. As $\Delta {\tilde{X}}_{k}$ increases, $\alpha_{k}$ decreases.

(2) The three-segment function model has two hyperparameters, $c_{\text{0}}$ and $c_{\text{1}}$, which serve as thresholds. When the error discrimination statistic is less than $c_{\text{0}}$, $\alpha_{k}=1$, and KF is performed. If the error discrimination statistic exceeds $c_{\text{1}}$, $\alpha_{k}=0$. When the error discrimination statistic is greater than $c_{\text{0}}$ but less than $c_{\text{1}}$, the weight of the state prediction model in state estimation is reduced. The functional form is as follows:


$$\alpha_{k}=\left\{ \begin{array}{cc} 1 & \left|\Delta {\tilde{X}}_{k}\right|\leq c_{\text{0}}\\ \frac{c_{\text{0}}}{\left|\Delta {\tilde{X}}_{k}\right|}{\left(\frac{c_{\text{1}}-\left|\Delta {\tilde{X}}_{k}\right|}{c_{\text{1}}-c_{\text{0}}}\right)}^{\text{2}} & c_{\text{0}}<\left|\Delta {\tilde{X}}_{k}\right|\leq c_{\text{1}}\\ 0 & \left|\Delta {\tilde{X}}_{k}\right|>c_{\text{1}} \end{array}\right.$$ (35)

In the Eq. (35), $c_{\text{0}}$ can be set between 1.0 and 1.5, and $c_{\text{1}}$ can be set between 3.0 and 4.5.

(3) The exponential function model uses a single hyperparameter as a threshold, and its functional form is as follows:


$$\alpha_{k}=\left\{ \begin{array}{cc} 1 & \left|\Delta {\tilde{X}}_{k}\right|\leq c\\ e^{-{\left(\left|\Delta {\tilde{X}}_{k}\right|-c\right)}^{\text{2}}} & \left|\Delta {\tilde{X}}_{k}\right|>c \end{array}\right.$$ (36)

In the Eq. (36), c is a constant with a value of 1.

**Fig 1:**
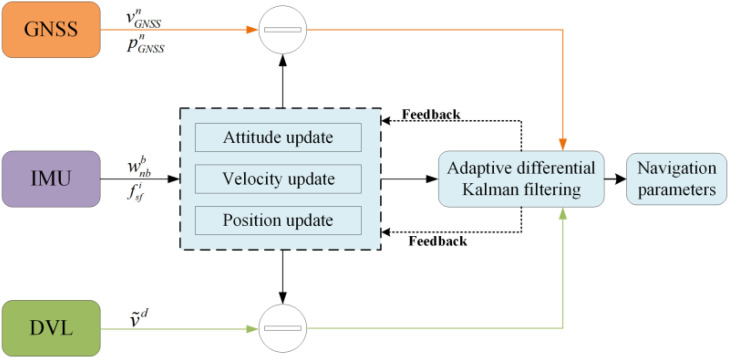
GNSS/SINS/DVL integrated navigation ADKF flow chart.

In a GNSS/SINS/DVL integrated navigation system, the accuracy of the state transition model is primarily determined by IMU errors. Generally, the IMU noise remains relatively stable, resulting in a highly accurate state transition model. Under such circumstances, the state transition model can be considered accurate, and the error discriminant statistics mainly reflect the measurement model errors. Therefore, an adaptive factor can be used to inflate the measurement noise covariance matrix, with the inflated noise replacing the nominal noise in the KF, thereby achieving robust handling of measurement noise. As shown in Fig 1, the diagram illustrates the specific procedure of adaptive robust filtering in GNSS/SINS/DVL integrated navigation.

### Experiments and analysis

#### Experimental procedure.

**Fig 2:**
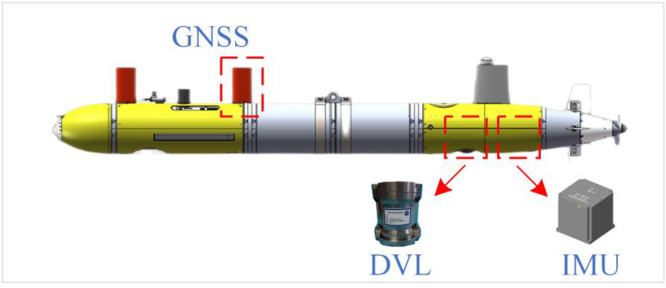
Experiment platform.

To validate the effectiveness of the ADKF algorithm for GNSS/SINS/DVL integration, this study conducted experiments using real-world UUV data collected at the Danjiangkou Reservoir in Nanyang, Henan, in August 2023. The experimental platform was the Orange Shark III-B UUV, as shown in Fig 2.

The UUV platform is equipped with a Beiyun A1 GNSS antenna, a CSSC Navigation Hailuo 98F IMU (fiber optic gyroscope and accelerometer), and a Nortek 300 kHz DVL. The detailed specifications of each sensor are provided in Table 1. The lever arm error between the GNSS antenna and the IMU is [0, 2.723, −0.558] m, while that between the DVL and the IMU is [0, 0.471, −0.044] m. The DVL exhibits a scale factor error of 2.55%, and the installation misalignment angles are [0.028, −0.010, −0.266].

**Table 1:** The main parameters of the sensor of Orange shark ⅢB UUV.

Sensor	Parameters	Numerical value
IMU	Sampling frequency	100 Hz
	Zero deflection of gyroscope	≤0.01${}^{\circ }/\text{h}$
	Random walk of angles	≤0.01${}^{\circ }/\text{h}$
	Zero bias of acceleration	≤1000μg
	Random walk of speed	≤100$\mu \text{g}/\sqrt{\text{Hz}}$
DVL	Sampling frequency	1 Hz
	Long-term accuracy	±0.1%±0.1 cm/s
	Single ping standard deviation	0.5 cm/s
GNSS	Sampling frequency	1 Hz

**Fig 3:**
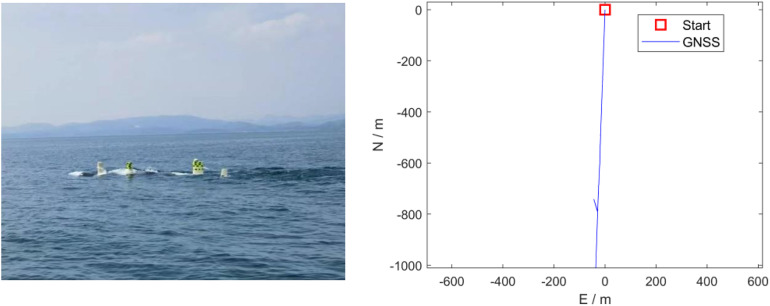
UUV experiment scenario and long line trajectory.

During the experiment, the team set up a GNSS base station on the shore and ensured that the UUV’s GNSS antenna remained above the water surface throughout its navigation. The PPP/SINS integrated navigation results calculated using Inertial Explorer (IE) 8.90 software were used as the reference baseline. For validation, a 2400-second segment of the navigation trajectory, covering approximately 4011 m along a long straight path, was selected. The experimental scenario and the straight trajectory are shown in Fig 3.

### Verification of measured data

To further analyze the comprehensive performance of the integrated navigation system, this study performs single point positioning (SPP) calculations on the GNSS observation data collected by the UUV using the RTKLIB software. The observation data adopt dual-frequency ionosphere-free combined pseudorange observations from the global positioning system (GPS), beidou navigation satellite system (BDS), galileo navigation satellite system (Galileo), and global navigation satellite system (GLONASS); the satellite cutoff elevation angle is set to 12°, and the tropospheric delay error is corrected using the Saastamoinen model. In the experiment, the post-processed navigation results of PPP/SINS (IE v8.90) are employed as the absolute reference datum, and the experimental trajectory data are respectively subjected to calculation and comparative analysis using the multi-sensor integrated navigation scheme and the single-sensor navigation scheme.

**Fig 4:**
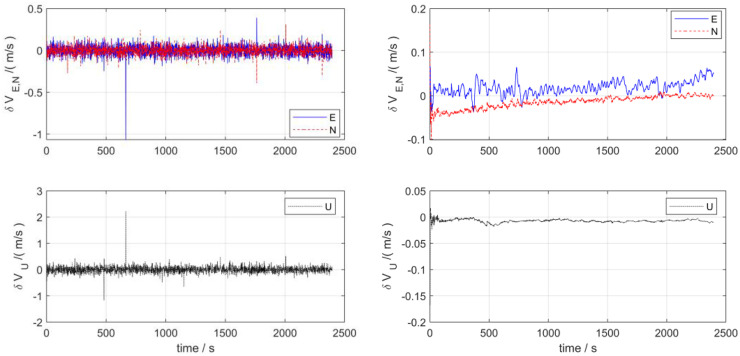
Velocity error in lake test.

Fig 4 presents the velocity error estimation curves of the two navigation schemes. As can be observed from the figure, for the navigation system relying solely on a single sensor, its velocity error exhibits significant large-amplitude oscillations and even pulse-like mutation characteristics, with poor stability. Whereas under the multi-sensor integrated navigation architecture, the time-varying characteristics of the velocity error tend to be significantly stable, the fluctuation amplitude is greatly suppressed, and the stability of velocity estimation in dynamic navigation scenarios is substantially improved.

**Fig 5:**
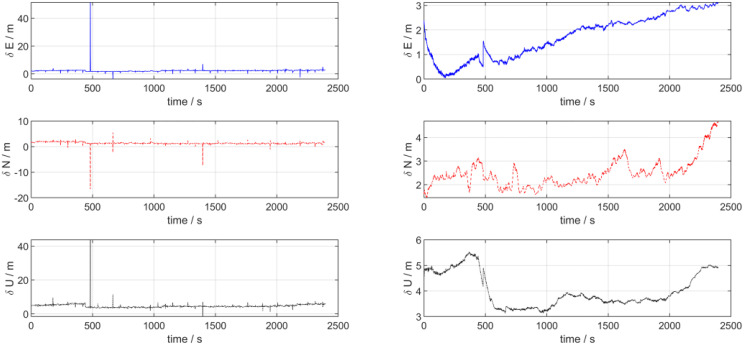
Position error in lake test.

Fig 5 presents the position error estimation curves of the two navigation schemes. As can be analyzed from the figure, the single-sensor-based navigation system exhibits distinct abrupt jumps or pulse-like sudden characteristics in position error (e.g., the eastward position error shows significant sharp peaks, while the northward position error demonstrates valley-like abrupt changes), indicating extremely unstable error behavior. In contrast, under the multi-sensor integrated navigation architecture, the position error exhibits a continuous and smooth evolution trend, which effectively suppresses the abrupt errors and cumulative effects that are prone to occur in single-sensor navigation.

Table 2 presents the quantitative statistics of velocity and position errors for the two schemes via the root mean squared error (RMSE). The results indicate that in the dynamic surface navigation and positioning scenario of the UUV, compared with the single-sensor navigation scheme, the multi-sensor integrated navigation exhibits superior performance in terms of the stability and accuracy of velocity and position errors by virtue of the multi-source sensor information fusion mechanism, and is more adaptable to the application requirements of dynamic navigation.

**Table 2:** Statistics of navigation parameters RMSE.

Scheme	Speed error/(m/s)			Positional error/(m)		
	E	N	U	E	N	U
GNSS	0.0508	0.0440	0.1011	2.3844	2.8315	4.7342
GNSS/SINS/DVL	0.0241	0.0205	0.0086	1.9244	2.5408	4.0566

To further evaluate the impact of the KF and the ADKF on integrated navigation solutions, gross errors were artificially introduced into the observation data in this experiment. A chi-square test was used to detect these gross errors, and the equivalent weight matrix of the measurement vector was calculated using the IGG scheme. Meanwhile, predicted residual statistics were constructed, and adaptive factors were calculated using two-segment, three-segment, and exponential function models to achieve better filtering performance. The dynamic positioning accuracy of SPP is approximately 3 m, and the normal operational noise of the DVL is about 0.1 m/s; accordingly, the initial value of the measurement noise covariance matrix was set to $diag(0.1\text{,0.1 ,0.1 ,5 ,5 , 5}{)}^{\text{2}}$. The collected data were processed as follows: during epochs 0 ~ 500, an acceleration of 0.5 $m/s^{\text{2}}$ was added to the inertial sensor’s acceleration data to simulate abnormal motion disturbances; during epochs 500 ~ 2400, random gross errors ranging from 1 to 60 m were added to the pseudorange observations, while random gross errors between 0.5 and 1 m/s were introduced into the velocity observations to simulate measurement anomalies. The following three filtering methods were then applied for solution computation:

Scheme 1: The adaptive factor is calculated using a two-segment function model, and the trajectory is processed separately with the KF and ADKF. The detection threshold for observation gross errors and abnormal motion disturbances is set to c=1.

Scheme 2: The adaptive factor is calculated using a three-segment function model, and the trajectory is processed separately with the KF and ADKF. The detection thresholds for observation gross errors and abnormal motion disturbances are set to $c_{\text{0}}=1.5,c_{\text{1}}=4$.

Scheme 3: The adaptive factor is calculated using an exponential function model, and the trajectory is processed separately with the KF and ADKF. The detection threshold for observation gross errors and abnormal motion disturbances is set to $c_{\text{2}}=1$.

**Fig 6:**
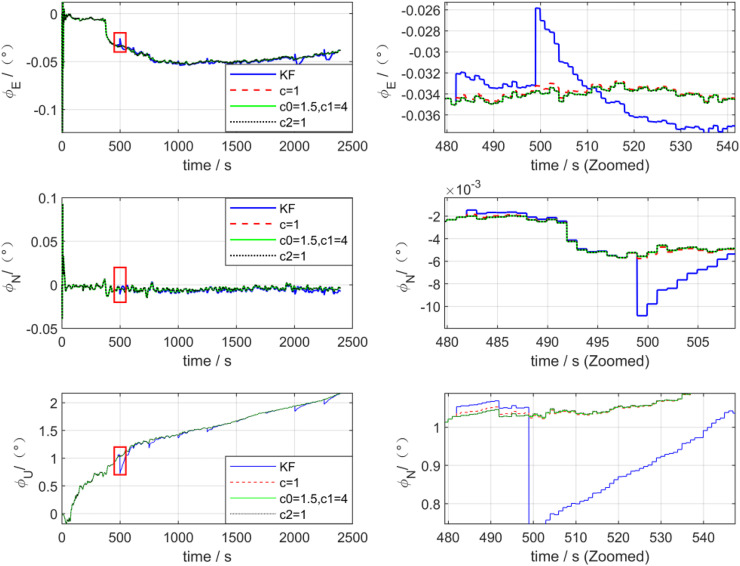
Attitude error of KF and ADKF processing.

Fig 6 presents the attitude error estimation curves processed by the two algorithms. As can be observed from the figure, after introducing outliers, in the east-north (E-N) direction, the maximum misalignment angles processed by the KF algorithm are −0.06° and −0.01°, respectively, while those processed by the ADKF algorithm are −0.05° and −0.005°, respectively. The estimation accuracies of the two algorithms for the upward misalignment angle are basically consistent. During the disturbance anomaly period (marked by the red box), the attitude error of KF exhibits a pulse-like mutation characteristic with severe transient error fluctuations; in contrast, the attitude error of ADKF evolves more smoothly without significant mutation phenomena, and exhibits a faster stable convergence trend afterward. The local enlarged view further verifies that the transient peak values of the attitude error of KF are significantly higher than those of ADKF, indicating that ADKF possesses superior anti-mutation and fast convergence capabilities against transient disturbances.

**Fig 7:**
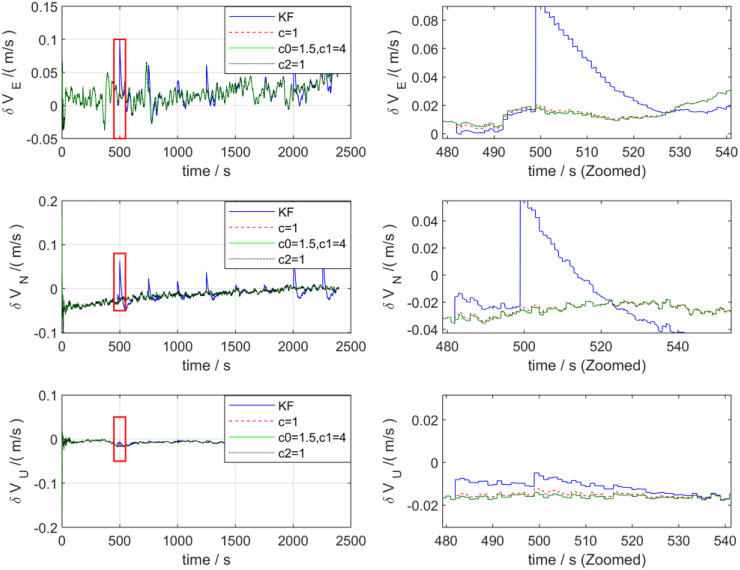
Speed error of KF and ADKF processing.

Fig 7 presents the velocity error estimation curves processed by the two algorithms. As can be observed from the figure, after introducing outliers, in the east-north (E-N) direction, the maximum velocity errors processed by the KF algorithm are 0.1 m/s and 0.8 m/s, respectively, while those processed by the ADKF algorithm are 0.05 m/s and 0.025 m/s, respectively, exhibiting a significant outlier suppression effect. The upward velocity fluctuates within the range of −0.02 to 0 m/s, and the estimation accuracies of the two algorithms for the upward velocity are basically consistent. Regarding the velocity error, KF exhibits large-amplitude sharp peak oscillations during the disturbance anomaly period, with distinct explosive characteristics of transient errors; in contrast, the velocity error of the ADKF shows almost no severe fluctuations during this period, remaining constrained within a narrow range, and possesses a faster real-time convergence speed. This characteristic demonstrates that ADKF has stronger robustness against real-time velocity disturbances and can effectively suppress the outbreak and propagation of transient errors.

**Fig 8:**
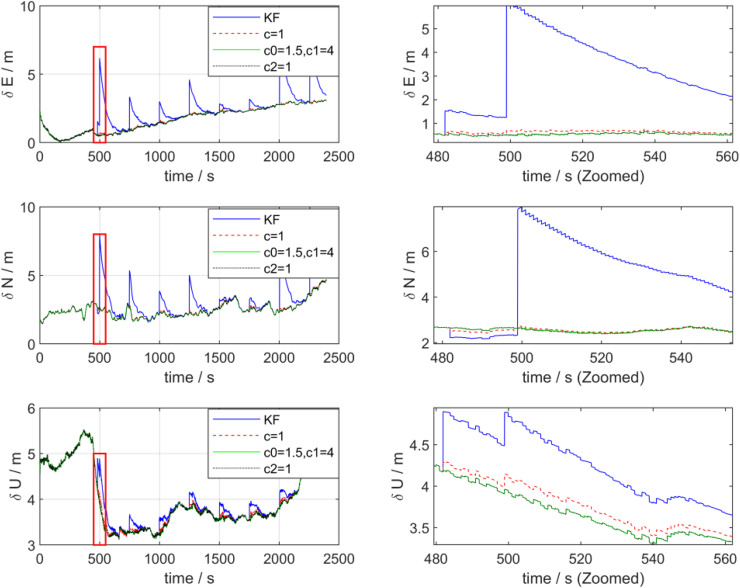
Position error of KF and ADKF processing.

Fig 8 presents the position error estimation curves processed by the two algorithms. As can be observed from the figure, after introducing outliers, the maximum deviations of the eastward and northward position errors processed by the KF algorithm both exceed 5 m, indicating that KF has weak resistance to outliers and exhibits numerous abrupt jumps. In contrast, the ADKF algorithm demonstrates a remarkable performance—especially at points with large outliers, ADKF can significantly mitigate the impact of outliers on positioning results. Additionally, the accuracy of the upward position processed by ADKF is also improved to a certain extent compared with that of KF. Regarding the position error, KF exhibits step-like abrupt changes during the disturbance anomaly period, with transient errors increasing rapidly and significantly in amplitude; in contrast, the position error of ADKF maintains a stable evolution trend, and the subsequent error accumulation trend is more moderate. The local enlarged view intuitively shows that ADKF can resist error sources (such as sensor anomalies, environmental disturbances, etc.) in real time, achieving more stable control and fast convergence of position errors.

Table 3 presents the statistical results of the root mean squared error (RMSE) for attitude, velocity, and position errors processed by the two filtering algorithms. From Table 3, it can be observed that the ADKF algorithm achieves varying degrees of improvement in attitude, velocity, and position accuracy compared with the KF algorithm. Specifically, the RMSE of the northward misalignment angle is decreased by 10.3%; the RMSE of the eastward velocity error is reduced by 7.7%, and that of the northward velocity error by 12%; the RMSE of the eastward position error is lowered by 28.1%, and that of the northward position error by 22.1%. In summary, by virtue of its adaptive robust mechanism, ADKF can dynamically adjust the filtering weights when responding to real-time disturbances, outliers, or sudden changes in sensor data, thereby suppressing the transient outbreak of errors and accelerating the convergence process. In contrast, KF is more sensitive to abnormal scenarios and prone to error mutations and transient loss of control. Based on the analysis of real-time error control and convergence characteristics from attitude and velocity to position, it can be concluded that ADKF exhibits stronger resistance to real-time disturbances, smoother error evolution, and faster convergence in the multi-sensor integrated navigation of UUV surface navigation. Consequently, ADKF possesses superior robustness and is more adaptable to the requirements of complex dynamic navigation scenarios.

**Table 3:** Statistics of navigation parameters RMSE.

Algorithm	Error Angle/(°)			Speed error/(m/s)			Positional error/(m)		
	E	N	U	E	N	U	E	N	U
KF	0.0434	0.0068	1.5068	0.0261	0.0233	0.0087	2.6649	3.2752	4.1198
Scheme 1	0.0427	0.0061	1.5068	0.0241	0.0205	0.0086	1.9353	2.5730	4.0570
Scheme 2	0.0427	0.0061	1.5067	0.0241	0.0205	0.0087	1.9154	2.5520	4.0386
Scheme 3	0.0427	0.0061	1.5068	0.0241	0.0205	0.0086	1.9155	2.5519	4.0417

## Conclusion

Based on the loosely coupled integrated navigation model in the structural domain, this study proposes ADKF algorithm that integrates the core technical advantages of observation epoch adjustment, KF, adaptive filtering, and robust filtering. By constructing a robust factor with superior robustness, the algorithm not only effectively enhances the filtering model’s capability for accurate identification and adaptive suppression of observation outliers but also significantly improves the algorithm’s dynamic adaptability to abnormal disturbances in carrier motion, essentially optimizing the stability and reliability of state estimation in the integrated navigation system. Compared with the loosely coupled model, the tightly coupled integrated navigation model based on the observation domain directly fuses GNSS pseudorange, carrier phase, and SINS/DVL raw measurement information. Theoretically, it possesses better information fusion efficiency and greater potential for precision improvement, and has become a core development direction of integrated navigation technology in the future. Therefore, subsequent research should focus on the in-depth fusion strategy of the adaptive robust mechanism and the tightly coupled model: first, design a hierarchical dynamic robust weight allocation scheme oriented to the heterogeneous error characteristics of multi-source sensors to achieve targeted suppression of multi-source observation outliers; second, construct a dynamic error covariance adaptive matching algorithm, optimize the fusion weight allocation of multi-source observation data by combining the idea of federated filtering, effectively address the key problems in the tightly coupled model such as the difficulty in accurately setting the initial value of the error covariance and the challenge of real-time tracking of time-varying errors, and fully tap the high-precision potential of the tightly coupled model.

## Supporting information

S1 File RAWdata. (ZIP)
